# Risk factors for dysplastic lesions in the proximal stomach in patients with familial adenomatous polyposis

**DOI:** 10.1055/a-2731-3533

**Published:** 2025-11-06

**Authors:** Hicham Bouchiba, Arthur S. Aelvoet, Barbara A.J. Bastiaansen, Patrick M.M. Bossuyt, Evelien Dekker

**Affiliations:** 1 Cancer Center Amsterdam, Department of Gastroenterology and Hepatology, University of Amsterdam, Amsterdam, the Netherlands; 2 Department of Epidemiology and Data Science, University of Amsterdam, Amsterdam, the Netherlands

**Keywords:** Endoscopy Upper GI Tract, Precancerous conditions & cancerous lesions (displasia and cancer) stomach, Diagnosis and imaging (inc chromoendoscopy, NBI, iSCAN, FICE, CLE), Endoscopy Lower GI Tract, Polyps / adenomas / ...

## Abstract

**Background and study aims:**

Gastric cancer is a recently recognized challenge in management of familial adenomatous polyposis (FAP), mostly developing in the proximal stomach amid carpeting fundic gland polyposis. Dysplastic lesions in the proximal stomach might be the precursor lesions for gastric cancer in FAP. We aimed to describe incidence of dysplastic lesions in the proximal stomach and to identify risk factors for these dysplastic lesions.

**Patients and methods:**

Data were collected from FAP patients who had undergone esophagogastroduodenoscopy (EGD) between 2015 and 2023 at our academic center. To identify putative risk factors for dysplastic lesions in the proximal stomach, we used multivariable Cox proportional hazard regression modeling.

**Results:**

Among the 196 FAP patients who underwent EGD at our center, 33 (17%) were diagnosed with dysplastic lesions in the proximal stomach. In total 61% were female and median age at diagnosis was 49 years (range 19–80). A total of 105 dysplastic lesions were identified. Ten (9.5%) showed high-grade dysplasia. During the study period, seven patients were diagnosed with gastric cancer. Carpeting fundic gland polyposis (≥ 100 polyps) (hazard ratio [HR] 8.94; *P* < 0.001), biliary reflux (HR 1.92; *P* = 0.017), and proton pump inhibitors (HR 1.78; *P* = 0.014) were significant predictors of dysplastic lesions in the proximal stomach. Advanced Spigelman stage (III/IV) (HR 0.37; *P* < 0.001) was associated with a significantly lower risk.

**Conclusions:**

Carpeting fundic gland polyposis, biliary reflux, and use of PPIs were identified as putative risk factors for dysplastic lesions in the proximal stomach. Presence of these risk factors should alert endoscopists to assess the stomach more thoroughly.

## Introduction

Familial adenomatous polyposis (FAP) is a rare inherited disorder resulting in formation of numerous adenomas in the colon and rectum. The disease is caused by a pathogenic variant in the adenomatous polyposis coli ( *APC* ) gene, which is located on chromosome 5 1 . If left untreated, these polyps will inevitably progress to colorectal cancer. Most patients, therefore, undergo prophylactic (procto)colectomy before age 30 2 . Patients with FAP may also develop extra-colonic disease, such as desmoid tumors and duodenal polyposis 3 .

Although in Japan a higher risk of gastric cancer in FAP patients was observed 4 5 , western patients were previously found not to be at greater risk compared with the general population 6 7 . A recent study reported a concerning increase in incidence of gastric cancer in western FAP patients 8 . In all described cases, gastric cancers arose in the proximal stomach and within areas of extensive fundic gland polyposis carpeting normal gastric mucosa. This extensive fundic gland polyposis might make it more difficult to detect and characterize gastric cancer and precursor lesions during endoscopy, highlighted by the worrisome fact that only two of 10 gastric cancers were endoscopically visualized, whereas the other eight were identified during random or targeted biopsies, during endoscopic ultrasound and prophylactic gastrectomy for what was thought to be high-grade dysplasia 8 9 . Fundic gland polyps seem to have a low but non-negligible risk of malignant progression, whereas up to one-third of them harbor low-grade dysplasia and 3% high-grade-dysplasia 10 .

**Fig. 1:**
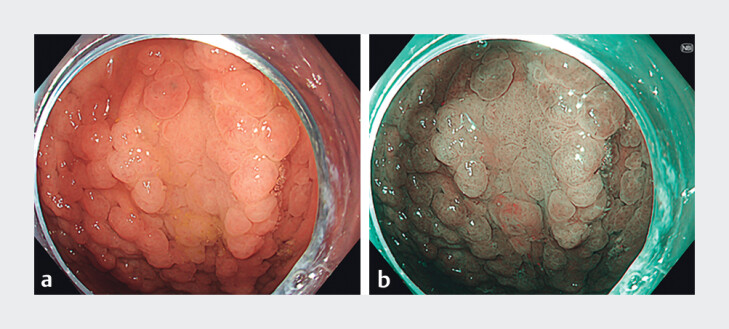
Dysplastic lesion with low-grade dysplasia in the corpus. **a** White appearance of a dysplastic field compared to surrounding mucosa. **b** With narrow-band-imaging, the demarcation between dysplasia and surrounding mucosa becomes clearer.

**Fig. 2:**
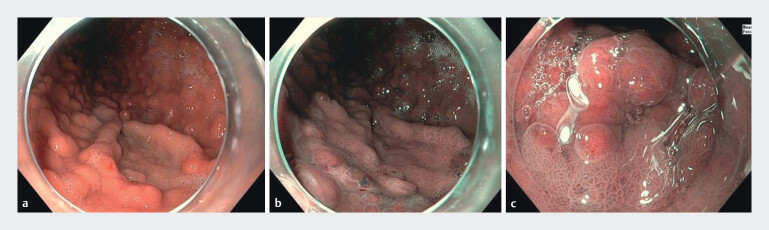
Dysplastic lesion with low-grade dysplasia in the corpus. **a** White appearance of a dysplastic field compared to the surrounding mucosa. **b** The same lesion visualized with narrow-band-imaging. **c** Near-focus imaging of the same lesion, showing a mucosal pattern that is distinguishable from the surrounding gastric mucosa and adjacent fundic gland polyps.

Gastric dysplastic lesions are thought to be precursor lesions for gastric cancer. Reported incidence of gastric dysplastic lesions in FAP varies widely around the globe, with higher numbers observed in Japan (15%-60%) 4 5 , while rarer in western countries (7%-29%) 11 12 . These might be true differences in incidence, but may also reflect a difference in the ability of endoscopists to detect gastric dysplasia, which might be extra challenging due to coexisting fundic gland polyposis. Fig. 1 and Fig. 2 illustrate two examples of proximal gastric dysplasia in FAP patients. Currently, there is no consensus on the optimal endoscopic surveillance strategy. Knowledge about factors associated with gastric dysplasia would be helpful to improve endoscopic surveillance and to identify patients at higher risk who should be examined more thoroughly during endoscopic surveillance.

The aim of this study was to describe endoscopic detection of dysplastic lesions in the proximal stomach and diagnosis of gastric cancer in our center in recent years. In addition, we aimed to identify putative risk factors for proximal gastric dysplastic lesions, because these may be precursor lesions for gastric cancer in FAP.

## Patients and methods

### Study group

We identified patients with FAP from our existing database in our center and retrospectively reviewed their eligibility during the study period: between January 2015 and August 2023. We selected this study period considering the recent shift in our focus from endoscopic surveillance of the duodenum and antrum to also the proximal stomach, aiming to improve detection of potential dysplastic lesions. Patients with a pathogenic variant of the *APC* gene or clinical presence of more than 100 colorectal adenomas with a positive family history of FAP were included if they had undergone at least one esophagogastroduodenoscopy (EGD).

Prior to data collection, patients provided informed consent for use of their data. This study was approved by the local Institutional Review Board and was conducted in accordance with the declaration of Helsinki.

### Data collection

We extracted the following data from patient records and endoscopy and pathology reports: age, sex, pathogenic variant of the *APC* gene, number of fundic gland polyps (categorized as 0, 1–50, 50–100, ≥ 100 and/or carpeting), *Helicobacter pylori* status, proton pump inhibitor (PPI) use, history of desmoid tumor, presence of biliary reflux, performed surgeries, and Spigelman stage. Carpeting fundic gland polyposis was defined as presence of 100 or more fundic gland polyps and/or with absence of any visible area of normal gastric mucosa in the proximal stomach. Biliary reflux was defined as visible bile in the stomach or biliary gastritis observed during upper endoscopy. Spigelman stage was determined by the number, size, and histology of duodenal polyps.

We only evaluated histologically confirmed gastric dysplastic lesions and cancers; cases diagnosed solely based on optical diagnosis were excluded. Dysplastic lesions were grouped by location: either distal in the antrum or proximal in the body or fundus of the stomach. Data on size, degree of dysplasia, and resection techniques were collected. Morphological characteristics of proximal dysplastic lesions and antral adenomas were classified based on endoscopic images and procedure reports. Proximal lesions were categorized as white mucosal patch, sessile lesion, polypoid mound, or pedunculated polyp, according to literature 13 . Antral adenomas were classified as either solitary sessile or centrally depressed lesions. Antral adenomas were not included in risk factor analysis, which focused exclusively on development of proximal gastric dysplasia.

### Statistical analysis

Our primary objectives were to describe incidence of dysplastic lesions in the proximal stomach of patients with FAP and to identify putative risk factors for these dysplastic lesions.

We calculated incidence of dysplastic lesions in the proximal stomach as the number of EGDs in which such a lesion was identified. Incidences were calculated from all EGDs performed per calendar year. Kaplan-Meier analysis was performed to describe cumulative incidence of gastric dysplastic lesions over the study period.

We performed multivariable Cox proportional hazards regression model to identify putative risk factors for dysplastic lesions in the proximal stomach. We calculated time from the first endoscopic surveillance to detection of a dysplastic lesion in the proximal stomach, if any, measured in months.

The following variables were considered as potential predictors: age, sex, number of fundic gland polyps, Spigelman stage, biliary reflux following duodenectomy, use of PPIs, and presence of desmoid.

We used the following variables as time-dependent variables in the Cox regression because only the information from the most recent EGD was used: number of fundic gland polyps (categorized as 0, 1–50, 50–100, ≥ 100 and/or carpeting), Spigelman stage, surgery performed (i.e. duodenal surgery), use of PPIs, and presence of desmoids. Each EGD was considered a separate timepoint in the Cox regression analysis and dysplastic lesions were evaluated per EGD. Therefore, unit of analysis in the regression was the number of dysplastic lesion positive EGDs, rather than number of patients. Because clinical characteristics such as age, surgery status, medication use, and Spigelman stage can change over time, we incorporated these as time-dependent variables using information from the most recent EGD.

We report hazard ratios (HRs) and corresponding 95% confidence intervals (CIs). *P* < 0.05 was considered to indicate statistically significant differences. Statistical analyses were performed using R version 4.2.1 (R Foundation for Statistical Computing, Vienna, Austria) using the packages haven and survival.

## Results

### Cohort

We were able to include data from all 196 eligible patients with FAP who had undergone at least one EGD between 2015 and 2023 at our center. The cumulative inclusion curve over the years is presented in Supplementary Fig. 1. In total, 118 histologically confirmed dysplastic lesions were identified: 79 antral adenomas and 105 proximal gastric dysplastic lesions. Antral adenomas were classified endoscopically as solitary sessile lesions (n = 43, 54.4%) or centrally depressed lesions (n = 34, 43.0%). Two antral adenomas (2.5%) harbored high-grade dysplasia. Of the 196 patients, 33 (17%) developed 105 dysplastic lesions in the proximal stomach. Median age at first diagnosis of a dysplastic lesion was 49 years. Table 1 summarizes baseline characteristics of the 196 patients included in this study, categorized by presence or absence of dysplastic lesions in the proximal stomach. Twenty of the patients diagnosed with dysplastic lesions were female (61%). Carpeting fundic gland polyposis was observed in 58% of patients diagnosed with a dysplastic lesion, compared with 12% of patients without a dysplastic lesion. Duodenal surgery was performed in 24% of patients diagnosed with a dysplastic lesion, compared with 13% of patients without dysplastic lesions.

**Table:** **Table 1** Baseline characteristics per patient and detection of dysplastic lesions in the proximal stomach.

	Proximal gastric dysplastic lesion(s) detected (n = 33)	No proximal gastric dysplastic lesion(s) detected (n = 163)
Median age in years (IQR)	48.8 (42.4–59)	45.8 (37.1–60.1)
Sex n (%)		
Female	20 (60.6%)	84 (51.5%)
Male	13 (39.4%)	79 (48.5%)
Pathogenic variant in *APC*	30 (93.8%)	137 (86.7%)
History of (procto)colectomy	30 (90.9%)	151 (92.6%)
Number of fundic gland polyps n (%)		
0	0	21 (12.9%)
1–50	4 (12.1%)	71 (43.6%)
50–100	10 (30.3%)	48 (29.4%)
≥ 100	19 (57.6%)	19 (11.7%)
No information	0	4 (2.5%)
History of *Helicobacter pylori* n (%)	0	7 (4.3%)
Proton pump inhibitor use n (%)	21 (63.6%)	85 (52.1%)
History of desmoid tumor n (%)	5 (15.2%)	19 (11.7%)
Spigelman stage n (%)		
0	2 (6.1%)	26 (16.0%)
I	3 (9.1%)	16 (19.8%)
II	13 (39.4%)	38 (23.3%)
III	7 (21.2%)	46 (28.2%)
IV	8 (24.2%)	37 (22.7%)
History of duodenal surgery n (%)	8 (24.2%)	21 (12.9%)
APC, adenomatous polyposis coli; IQR, interquartile range.		

### Characteristics of proximal gastric dysplastic lesions

Median size of the 105 dysplastic lesions was 18 mm, ranging from 1 to 100 mm. The majority appeared as white mucosal patches (n = 49, 46.7%), followed by sessile lesions (n = 33, 31.4%), polypoid mounds (n = 18, 17.1%), and pedunculated polyps (n=4, 3.8%). All but one of these lesions were removed by piecemeal endoscopic mucosal resection, whereas a 45-mm dysplastic lesion was treated by endoscopic submucosal dissection. Ten (9.8%) of these dysplastic lesions harbored high-grade dysplasia.

### Detection of dysplastic lesions in the proximal stomach

**Fig. 3:**
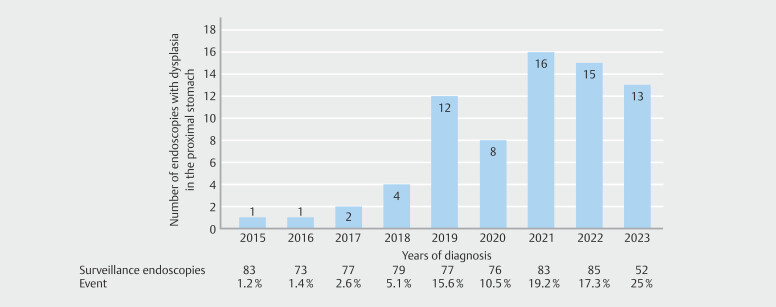
Detection of dysplastic lesions in the proximal stomach over the years.

**Fig. 4:**
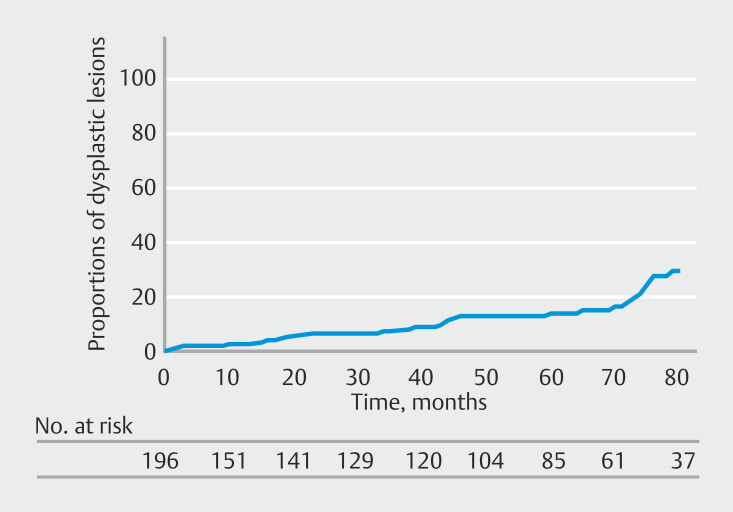
Cumulative incidences of dysplastic lesions in the proximal stomach.

A total of 685 EGDs were performed between 2015 and 2023 in the 196 included patients. During this period, patients had undergone a median of three surveillance endoscopies (range 1–12). Fig. 3 shows the number of EGDs that detected dysplastic lesions in the proximal stomach relative to the total number of surveillance endoscopies per calendar year. In 2015, the detection rate was 1.2%, which increased steadily in the following years. In the last 3 years, dysplastic lesions in the proximal stomach were detected in at least 17% of EGDs in FAP patients. Fig. 4 shows cumulative incidence of proximal gastric dysplastic lesions. At 5 years, cumulative incidence was 14.3%.

### Risk factors for dysplastic lesions in the proximal stomach

Cox proportional hazards regression analysis identified several variables as significant predictors for dysplastic lesions in the proximal stomach ( Table 2 ). Fundic gland polyps between 50 and 100 (HR 3.62; *P* = 0.004), carpeting fundic gland polyposis (HR 8.94; *P* < 0.001), biliary reflux following duodenectomy (HR.92; *P* = 0.017), and use of PPIs (HR 1.78; *P* = 0.014) were significantly associated with dysplastic lesions in the proximal stomach ( Table 2 ). In contrast, an advanced Spigelman stage (III/IV) (HR 0.37; *P* < 0.001) was associated with a lower risk of proximal dysplastic lesions.

**Table:** **Table 2** Cox proportional hazard regression analysis of development of proximal gastric dysplasia.

Variable	Group	Hazard ratio (HR)	95% CI (lower-upper)	*P* value
Age in years		0.99	0.977–1.013	0.58
Sex	Female	1.000 (Reference)		
	Male	0.95	0.604–1.478	0.81
Fundic gland polyposis	0–50	1.000 (Reference)		
	50–100	3.63	1.780–7.415	0.0004
	≥ 100	8.94	4.766–16.769	< 0.0001
Biliary reflux following duodenectomy	No	1.000 (Reference)		
	Yes	1.92	1.122–3.272	0.02
Presence of desmoid	No	1.000 (Reference)		
	Yes	1.05	0.606–1.813	0.87
Use of PPI	No	1.000 (Reference)		
	Yes	1.78	1.126–2.822	0.01
Spigelman classification	0, I and II	1.000 (Reference)		
	III and IV	0.37	0.229–0.599	< 0.001
CI, Confidence Interval; PPI, proton pump inhibitor.				

### Gastric cancer cases

During the study period, seven patients (3.6%) were diagnosed with gastric cancer. Six cases were located in the proximal stomach, all in patients with carpeting fundic gland polyposis, and all had previously undergone endoscopic resection of a proximal dysplastic lesion. However, it remains uncertain whether the cancers originated from the same site. Three cancers were found in lesions that appeared endoscopically as protruding areas suspicious for high-grade dysplasia or early cancer. Another case presented as an ulcerated lesion, including one diagnosed during emergency endoscopy for hematemesis. In the remaining two cases, gastric cancer was not endoscopically visible and was only diagnosed during autopsy following metastatic disease. Further clinical and histologic details of these cases have been described previously 14 .

## Discussion

In recent years, detection rates for dysplastic lesions in the proximal stomach in FAP patients at our center have increased. Putative risk factors for presence of these lesions include 50 to 100 fundic gland polyps, more than 100 fundic gland polyps, biliary reflux following duodenectomy, and use of PPIs. In contrast, an advanced Spigelman stage (III-IV) was associated with a significantly lower risk for dysplastic lesions in the proximal stomach.

Our study revealed a 17% incidence of dysplastic lesions in the proximal stomach.

A study from the UK and the Netherlands by Martin et al. reported an overall gastric adenoma rate of 14%, but a proximal gastric adenoma detection rate of only 5.5% 15 . Similarly, Ngamruenphong et al. found proximal gastric adenomas in just 5.2% of their American cohort 16 . These differences in incidence rates may be attributed to the older endoscopic surveillance cohorts in the studies by Martin et al. (1975–2015) and Ngamruenphong et al. (2004–2013) when compared with our recent cohort (2015–2023). Because an alarming rise in gastric cancer diagnosis has been reported in FAP patients, this may have led to increased awareness and detection of potential precursor lesions. When looking at Asia, Japanese studies have reported significantly higher incidences of gastric adenomas 4 5 . A recent multicenter study in Japan reported gastric adenomas in 24% of FAP patients 17 , but the location of these gastric adenomas was not described. The higher incidence of gastric cancer in FAP patients in Japan may be due to a possibly different etiology, in which dysplasia may be related to *H. pylori* infection, which is more common in Asia 18 . In our series, patients with proximal dysplastic lesions did not have a history of *H. pylori* infection.

We observed a significant increase in the detection rate of dysplastic lesions in the proximal stomach, from 1.2% in 2015 to 25% in 2023. This impressive increase could be attributed to increased awareness of gastric dysplastic lesions resulting from diagnosis of multiple patients with gastric cancer at our center and simultaneous reports in the literature 8 19 . Environmental factors such as changes in diet, lifestyle, and possibly increased exposure to carcinogens may also contribute to increased incidence of these dysplastic lesions and cancers 20 .

Carpeting fundic gland polyposis and having between 50 and 100 fundic gland polyps are putative risk factors for proximal gastric dysplastic lesions. Notably, all reported cases of proximal gastric cancer in the West and in our series involved patients with carpeting fundic gland polyposis 7 8 14 . However, it remains unclear whether these dysplastic lesions and cancers originate directly from fundic gland polyps or whether they develop independent of them.

In the series by Mankaney et al., visualization of gastric cancer was challenging, with only two of 10 cases being visualized endoscopically 8 . We propose that fundic gland polyps may contribute to the obscuring effect, making it more difficult to identify proximal gastric cancer and its potential precursor lesions. This observation may account for the lower detection rate in the earlier phase of our study. In patients with fundic gland polyps, use of advanced imaging techniques, such as narrow-band imaging, is recommended to improve detection and demarcation of dysplastic lesions in the proximal stomach, which are usually lighter than the background ( Fig. 1 and Fig. 2) 21 . In our center, we sometimes change patient position from left lateral to supine during endoscopic surveillance, which may improve visualization and detection of gastric dysplastic lesions. With the help of gravity, the space between overlying fundic gland polyps becomes visible, which may lead to detection of lesions that were not visible in the original left lateral position.

In addition, use of PPIs was identified as a putative risk factor for dysplastic lesions in the proximal stomach. PPIs may be linked to development of sporadic fundic gland polyps 22 23 24 . In FAP, development of fundic gland polyps is due to the pathogenic variant of APC 25 , but the exact mechanism of PPI-induced fundic polyp development is not fully understood. It is hypothesized that gastric acid suppression may stimulate gastrin production, subsequently causing parietal cell hyperplasia and hypertrophy 26 . However, Khalaf et al. found no association between fundic gland polyps and gastric acidity levels 27 . Fukuda et al. performed a histological analysis of sporadic fundic gland polyps in PPI users, noting larger size and foveolar hyperplasia, although dysplasia was not examined 28 . Although a previous study suggested a potential protective effect of acid-suppressive therapy (including both proton pump inhibitors and H _2_ -receptor antagonists) against dysplasia in FAP, this was based on a relatively small cohort of 75 patients 10 . In our study, we specifically examined PPI use, which may be more common in patients with extensive fundic gland polyposis. Because fundic gland polyposis itself is associated with dysplasia, the observed association with PPI use may be indirect.

Another putative risk factor for dysplastic lesions in the proximal stomach was biliary reflux following duodenectomy. We hypothesize that bile exposure may lead to chronic inflammation resulting in biliary gastritis, which in combination with a pathogenic variant in the *APC* gene, may act as a driver for dysplasia. This aligns with findings by Spigelman et al., who observed an association between duodenogastric bile reflux and gastric adenomas 29 . Sobala et al. also demonstrated a positive association between bile reflux, reactive gastritis, and intestinal metaplasia 30 . Therefore, it is crucial to clear the stomach of biliary fluids and to carefully assess it for potential mucosal changes during endoscopic examinations.

Interestingly, an advanced Spigelman stage of III-IV was significantly associated with a lower risk of dysplastic lesions in the proximal stomach. This inverse association may reflect a historical focus on duodenal surveillance in these patients, potentially missing early gastric lesions. Moreover, we hypothesize that proximal (pre)cancerous lesions in the stomach may differ and are not correlated with the severity of duodenal involvement. Two studies support these findings, because they did not find any differences between Spigelman stages 16 31 . Therefore, endoscopic surveillance strategies should include not only the duodenum but also the stomach, and the European FAP consortium has introduced such a novel surveillance protocol for the upper gastrointestinal tract 32 . As part of this approach, lesions that appear suspicious, for example, due to an altered mucosal pattern or whitish appearance and that measure ≥ 5 mm are resected during surveillance.

This study is the largest to date for identifying risk factors for dysplastic lesions in the proximal stomach in FAP patients. However, due to its historical design, certain limitations are inevitable. It is possible that data for each patient in our electronic patient system, such as use of PPIs, were not consistently updated. Moreover, included gastric dysplastic lesions were not further specified based on histology. The most common described gastric dysplastic lesions in FAP based on histology include foveolar type adenomas, pyloric gland adenomas, intestinal type adenomas, and fundic gland polyps with dysplasia 12 33 . It might be that these lesions have a different risk of malignant transformation. White mucosal patches are among the most frequently observed endoscopic appearances of proximal gastric dysplasia in FAP patients 21 34 35 . Dysplastic gastric lesions can have different endoscopic appearances, which might be associated with different histology. A recent study demonstrated that, based on endoscopic appearances, different subtypes of gastric lesions can be distinguished, which correlate with underlying histologic subtypes 35 . Further research is essential for accurate endoscopic and histologic classification of gastric lesions in FAP and for identification of potential precursor lesions of gastric cancer.

## Conclusions

Although incidence of colorectal and duodenal cancer in FAP has decreased in recent decades, detection of gastric cancer and dysplasia seems to have increased. We identified several readily available variables as putative risk factors for dysplastic lesions in the proximal stomach. Whether these are genuine risk factors should be evaluated in future cohorts. If so, they may guide endoscopists toward more thoroughly scoping patients with these risk factors, potentially leading to detection of dysplastic lesions. Challenges of detecting dysplastic lesions in the proximal stomach, particularly with the obscuring effect of carpeting fundic gland polyposis, clearly emphasize the need for high-quality endoscopic surveillance in FAP expert centers.

## Supplementary Material

Supplementary Material Zusatzmaterial
