# Supplementary figures and images for: Risk factors for dysplastic lesions in the proximal stomach in patients with familial adenomatous polyposis
Source: Endosc Int Open. 2025 Nov 6;13:a27313533. doi: 10.1055/a-2731-3533 (PMC12596990; doi:10.1055/a-2731-3533)

**Supplementary Fig. 1** Cumulative inclusion over the study period.

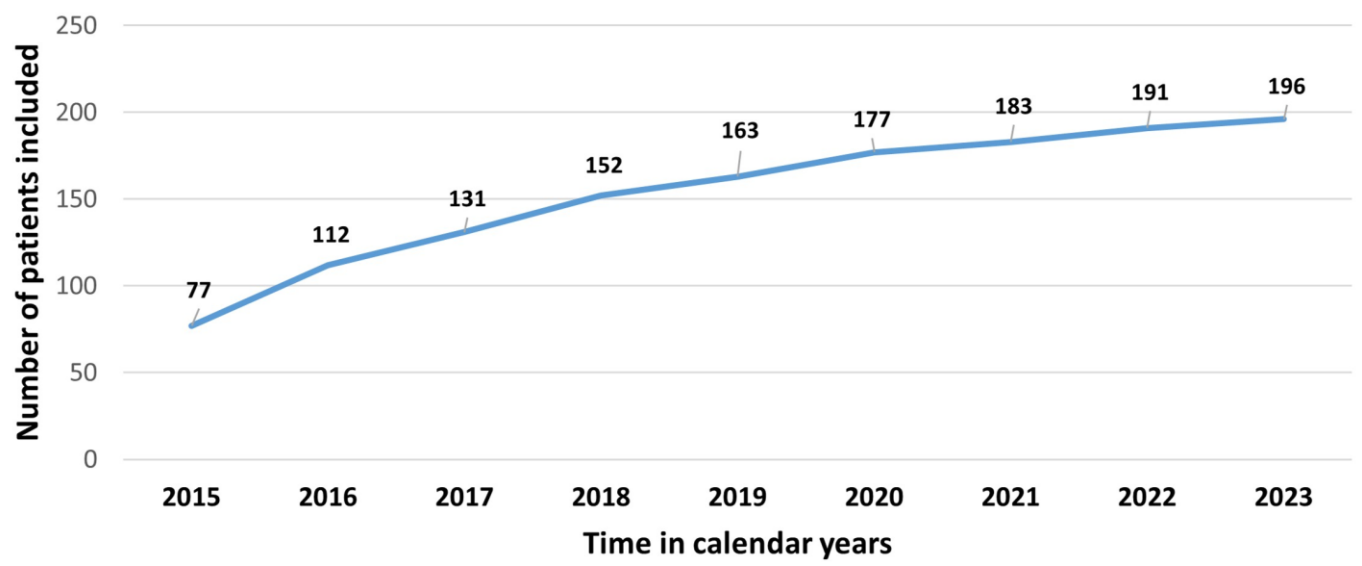

Supplement: Supplementary file 1 — Supplementary Material [file 10-1055-a-2731-3533_27337548.pdf]
